# Nuclei Isolation Methods on Frozen Clotted Blood Samples

**DOI:** 10.21769/BioProtoc.5573

**Published:** 2026-01-20

**Authors:** Melissa Cuevas, Kenneth Jones, Nancy Hadley Miller

**Affiliations:** 1 Department of Orthopedics, University of Colorado Anschutz Medical Campus, Aurora, CO, USA; 2 Musculoskeletal Research Center, Children’s Hospital Colorado, Aurora, CO, USA

**Keywords:** Nuclei dissociation, Frozen blood samples, Clotted blood, B cells, T cells, Flow cytometry

## Abstract

It is common practice for laboratories to discard clotted blood or freeze it for future DNA extraction after extracting serum from a serum-separating tube. If freezing for DNA extraction, the blood clot is not usually cryopreserved, which leads to cell membrane fragility. In this protocol, we describe steps to isolate high-quality nuclei from leukocytes derived from whole blood samples frozen without a cryoprotective medium. Nuclei isolated from this protocol were able to undergo ATAC (assay for transposase-accessible chromatin) sequencing to obtain chromatin accessibility data. We successfully characterized and isolated B cells and T cells from leukocytes isolated from previously frozen blood clot using Miltenyi’s gentleMACS Octo Dissociator coupled with flow sorting. Nuclei showed round, intact nuclear envelopes suitable for downstream applications, including bulk sequencing of nuclei or single-cell nuclei sequencing. We validated this protocol by performing bulk ATAC-seq.

Key features

• This protocol is compatible with previously collected blood that has been frozen.

• Previous cryopreservation of the samples is not required for this protocol.

• This protocol enables flow sorting of non-viable leukocytes for a more precise cell population for bulk sequencing experiments.

## Graphical overview


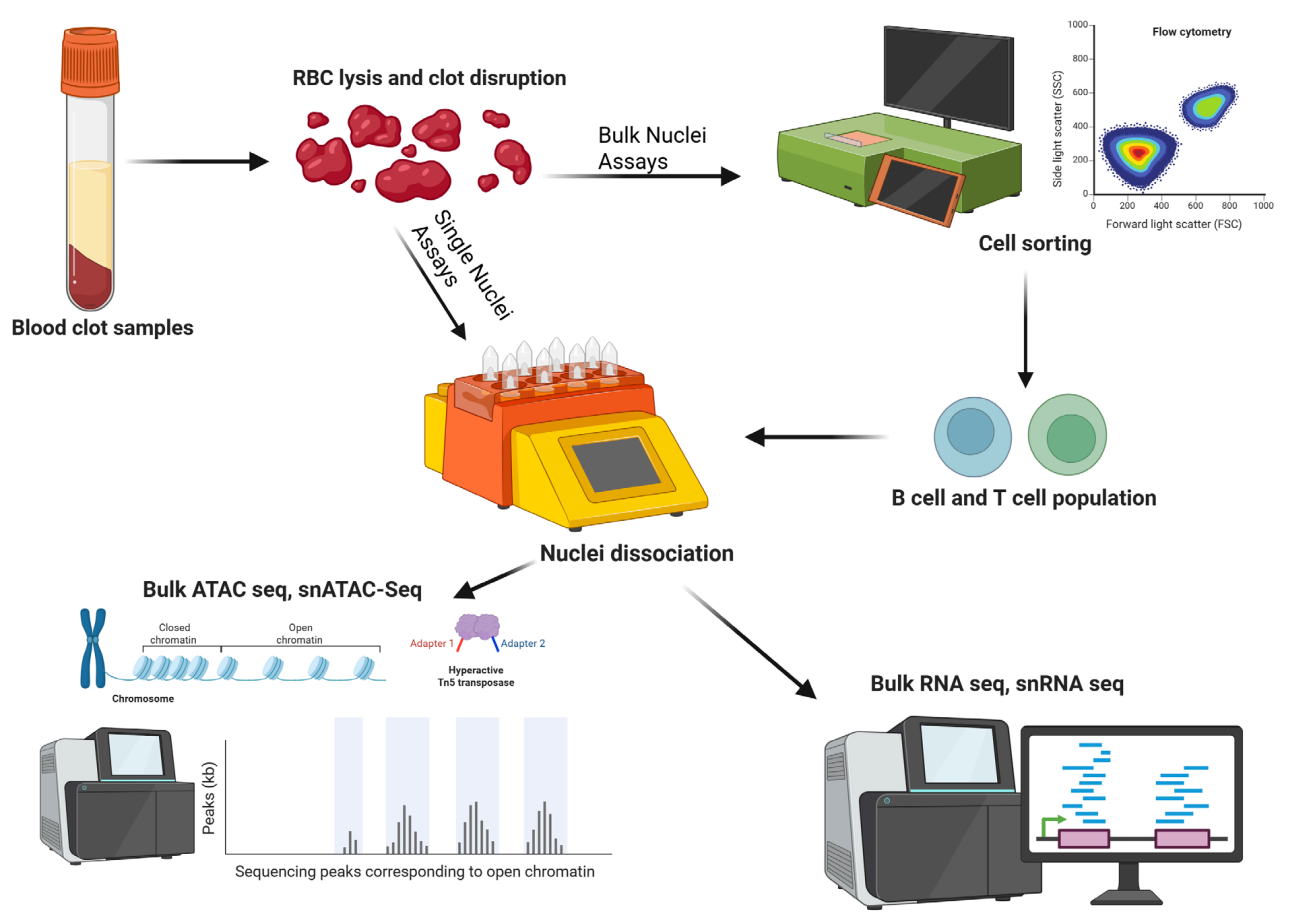


## Background

Biobanks are an essential tool in human disease research. Blood samples are ubiquitous in biobanks due to their versatility and ease of collection in clinical settings [1]. Technological advancements in biomedical research continue to elucidate the molecular mechanisms of diseases. However, many biobanks in existence predated these technologies [2–4]; samples are often collected with unspecific future assays in mind, and optimal preservation methods were not always known at the time of collection, especially for samples collected prior to newer technical advances. Blood samples kept in -80 °C freezers without cryoprotectants lose cellular integrity but often have some viable nucleic acids that can be extracted. One common strategy is to extract all the nucleic acids from the entire sample to perform DNA or RNA sequencing [5,6]. While this is useful, all epigenetic modifications are stripped from the DNA, effectively shutting out epigenetic studies from historical samples. Obtaining total mRNA is also challenging with these samples due to high endogenous RNase activity from cell lysis that occurs during freezing without cryoprotectants [7]. The ability to extract nuclei from these samples enables researchers to obtain more data for disease mechanisms with genetic components.

Nuclei-based assays are becoming more popular in the study of molecular mechanisms [8]. Researchers are increasingly investigating variable genetic expression related to diseases by integrating multiple datasets for a more holistic, multiomics approach. The assay for transposase-accessible chromatin using sequencing (ATAC sequencing) is a useful method for mapping chromatin accessibility. Bulk ATAC sequencing and single-nuclei ATAC sequencing both require good-quality nuclei as input, as opposed to whole cells [9–11]. Nuclei can also serve as input for nuclear RNA sequencing and single-nuclei RNA sequencing [12–14]. Even though nuclei sequencing ignores mRNA present in the cytoplasm, important transcriptomic data can still be gathered from samples that are difficult to dissociate into a single-cell suspension or samples with compromised cell membranes.

Here, we describe a protocol to extract nuclei from leukocytes from frozen blood samples. This protocol is useful for applications where blood samples were previously frozen without any cryopreservation or PBMC isolation. It is compatible with fresh blood clot samples in a serum-separating tube, which is incompatible with Ficoll gradient due to the clotting factor present [15]. Additionally, we show that the leukocyte extraction protocol is gentle on fragile cells, allowing cell sorting to be done by flow cytometry prior to nuclei extraction. This enables bulk sequencing assays from a purified population of nuclei, which may be more cost-effective than single-nuclei assays. The flexibility of this protocol makes it adaptable to both frozen and fresh clotted blood, and can help researchers gather valuable data from older, previously banked samples that may still have intact nuclei and mostly intact cell membranes. Flow sorting is successful despite the non-viability of the cells after leukocyte extraction.

This protocol has been validated using frozen blood clot, including flow cytometry and bulk ATAC-seq. Performing flow cytometry steps is only necessary if bulk sequencing is the end goal to isolate nuclei from a homogeneous population. This part of the protocol can be omitted if the endpoint experiment is a single-nuclei assay. We have reliably isolated >700,000 nuclei from one tube of frozen blood clot that was left from an 8.5 mL serum-separating tube after serum was extracted. The volume of blood clot typically left behind is approximately 3 mL. This protocol has also been successfully conducted on fresh clotted blood by adjusting lysis times and running the 4C_Nuclei_1 dissociation protocol twice on a gentleMACS Octo Dissociator. It may be possible to apply this protocol to frozen unclotted blood in EDTA or sodium heparin blood tubes to eliminate the Ficoll gradient step; however, we did not verify these sample types. Miltenyi Biotec product sheets were used as a reference for sections C and D [16,17], but optimization was done for this specific sample type.

## Materials and reagents

**Biological materials**

1. Frozen blood clot

2. Fresh blood clot (optional)

**Reagents**

1. Bovine serum albumin (BSA) (MilliporeSigma, catalog number: 12660910GM)

2. Flow cytometry antibodies (only applicable if flow sorting)

a. FITC CD3 antibody (Miltenyi Biotec, catalog number: 130-113-700)

b. PE CD19 antibody (Miltenyi Biotec, catalog number: 130-114-172)

c. VioBlue CD11B (Miltenyi Biotec, catalog number: 130-110-616)

d. APC CD11C (Miltenyi Biotec, catalog number: 130-114-110)

e. Other antibodies (optional): Add other suitable antibodies to tailor the cell population of interest

3. Compensation beads (Invitrogen, catalog number: 01-2222-41)

4. Anti-nucleus microbeads (Miltenyi Biotec, catalog number: 130-132-997)

5. RNase inhibitor (Applied Biosystems, catalog number: N8080119)

6. Ethanol 200 proof (Decon, catalog number: 2701)

7. Puregene RBC lysis solution (Qiagen, catalog number: 158106)

8. EDTA (Lonza, catalog number: 51201)

9. Phosphate-buffered saline (PBS) (Gibco, catalog number: 10010023)

10. Nuclei extraction buffer (Miltenyi Biotec, catalog number: 130-128-024)

11. Trypan Blue solution, 0.4% (Gibco, catalog number: 15250061)

**Solutions**

1. 1% BSA (see Recipes)

2. MACS buffer (see Recipes)

3. Lysis buffer (see Recipes)

4. Nuclei separation buffer (see Recipes)

5. Resuspension buffer (see Recipes)

**Recipes**

*Note: All total volumes are per sample.*

**1. 1% BSA**

**Table:** 

Reagent	Final concentration	Quantity or volume
BSA	n/a	10 mg
PBS	n/a	Bring final volume to 1 mL

**2. MACS buffer**

**Table:** 

Reagent	Final concentration	Quantity or volume
1× PBS	n/a	9.96 mL
BSA	10 mg/mL	100 mg
0.5 M EDTA	2 mM	40 μL
Total	n/a	10 mL

**3. Lysis buffer**

**Table:** 

Reagent	Final concentration	Quantity or volume
RNase inhibitor	0.2 U/μL	40 μL
Nuclei extraction buffer	n/a	3.96 mL
Total	n/a	4 mL

**4. Nuclei separation buffer**

**Table:** 

Reagent	Final concentration	Quantity or volume
RNase inhibitor*	0.2 U/μL	80 μL*
1% BSA	0.04%	320 μL
Nuclei extraction buffer	14%	1.12 mL
PBS	n/a	6.56 mL or 6.48 mL*
Total	n/a	8 mL

*Add RNase inhibitor to this buffer if RNA sequencing is desired as a downstream application.

Adjust PBS volume as necessary.

**5. Resuspension buffer**

**Table:** 

Reagent	Final concentration	Quantity or volume
RNase inhibitor	0.2 U/μL	15 μL
1% BSA	0.1%	1.5 μL
PBS	n/a	1.48 mL
Total	n/a	1.5 mL

**Laboratory supplies**

1. Serum-separating tube 8.5 mL (BD Vacutainer, catalog number: 0268396)

2. Serological pipettes 5 mL (Fisherbrand, catalog number: 1367811D)

3. Serological pipettes 10 mL (Fisherbrand, catalog number: 1367811E)

4. Serological pipettes 25 mL (Fisherbrand, catalog number: 1367811)

5. TipOne RPT filter tips P1000 (USA Scientific, catalog number: 1182-1830)

6. TipOne RPT filter tips P200 (USA Scientific, catalog number: 1180-8810)

7. TipOne RPT filter tips P20 (USA Scientific, catalog number: 1180-1810)

8. TipOne RPT filter tips P10 (USA Scientific, catalog number: 1182-3810)

9. Kimwipes (KimClark, catalog number: 066-66A)

10. LS columns (Miltenyi Biotec, catalog number: 130-042-401)

11. QuadroMACS separator (Miltenyi Biotec, catalog number: 30-090-976)

12. gentleMACS C tubes (Miltenyi Biotec, catalog number: 130-093-237)

13. MTC^TM^ Bio Screw Cap MacroTubes^®^ 5 mL (MTC Bio, catalog number: MTCC2545)

14. 15 mL conical tubes (Nunc, catalog number: 12-656-269)

15. 50 mL conical tubes (Nunc, catalog number: 12-565-271)

16. Microcentrifuge tubes (Thermo Fisher, catalog number: 3541)

17. Cryovials (Genesee Scientific, catalog number: 24-202P) (only for optional freezing of leukocyte aliquots)

18. MACS SmartStrainers 70 μm (Miltenyi Biotec, catalog number: 130-098-462)

19. MACS SmartStrainers 30 μm (Miltenyi Biotec, catalog number: 130-098-458)

20. 5 mL Polyprene round-bottom tube (Falcon, catalog number: 352063) (only for flow sorting with drop-based cell sorters such as Sony MA900)

21. HighSpeed MACSQuant Tyto cartridge (Miltenyi Biotec, catalog number: 130-121-549) (only required if using Miltenyi MACSQuant Tyto Cell Sorter)

## Equipment

1. Water bath

2. Centrifuge with cooling capability that accommodates 15 and 50 mL conical tubes (Beckman Coulter Allegra X-30 or equivalent)

3. Microcentrifuge with cooling capability (Sorvall™ Legend™ Micro 17R Microcentrifuge, catalog number: 75002440 or equivalent)

4. Vortexer

5. Rocker (Benchmark Scientific, catalog number: BR2000 or equivalent)

6. Serological pipette controller (Eppendorf, catalog number: 4430000018 or equivalent)

7. Micropipettes (Eppendorf, catalog number: 2231001168 or equivalent)

8. gentleMACS Octo Dissociator (Miltenyi Biotec, catalog number: 130-095-937)

9. gentleMACS Octo Coolers (Miltenyi Biotec, catalog number: 130-130-533)

10. MACS MultiStand (Miltenyi Biotec, catalog number: 130-042-303)

11. Cell Sorter

*Note: This protocol has been verified using both the Miltenyi MACSQuant Tyto Cell Sorter and the Sony MA900 Multi Application Cell Sorter.*

## Software and datasets

1. Adobe PDF (version 2025.001.20997)

2. Photoviewer (for microscopy image viewing)

*Note: All gentleMACS Octo dissociator programs used in this protocol were pre-programed into the device. We did not modify any programs. The Multi_A_01 protocol used on the gentleMACS Octo Dissociator takes 36 s in total duration, with 268 total rounds per run. The 4C_nuclei_1 takes 5 min and 15 s in total duration, with 2415 total rounds per run. Both devices used for flow cytometry export results as PDF.*

## Procedure

**A. Leukocyte extraction from frozen clotted whole blood**

*Note: Prior to starting, cool the RBC lysis solution by placing it on ice, cool the centrifuge to 4 °C, and set the water bath to 37 °C. Keep samples and reagents cold throughout the protocol. All centrifugation steps are done at 4 °C.*

**Figure 1.**
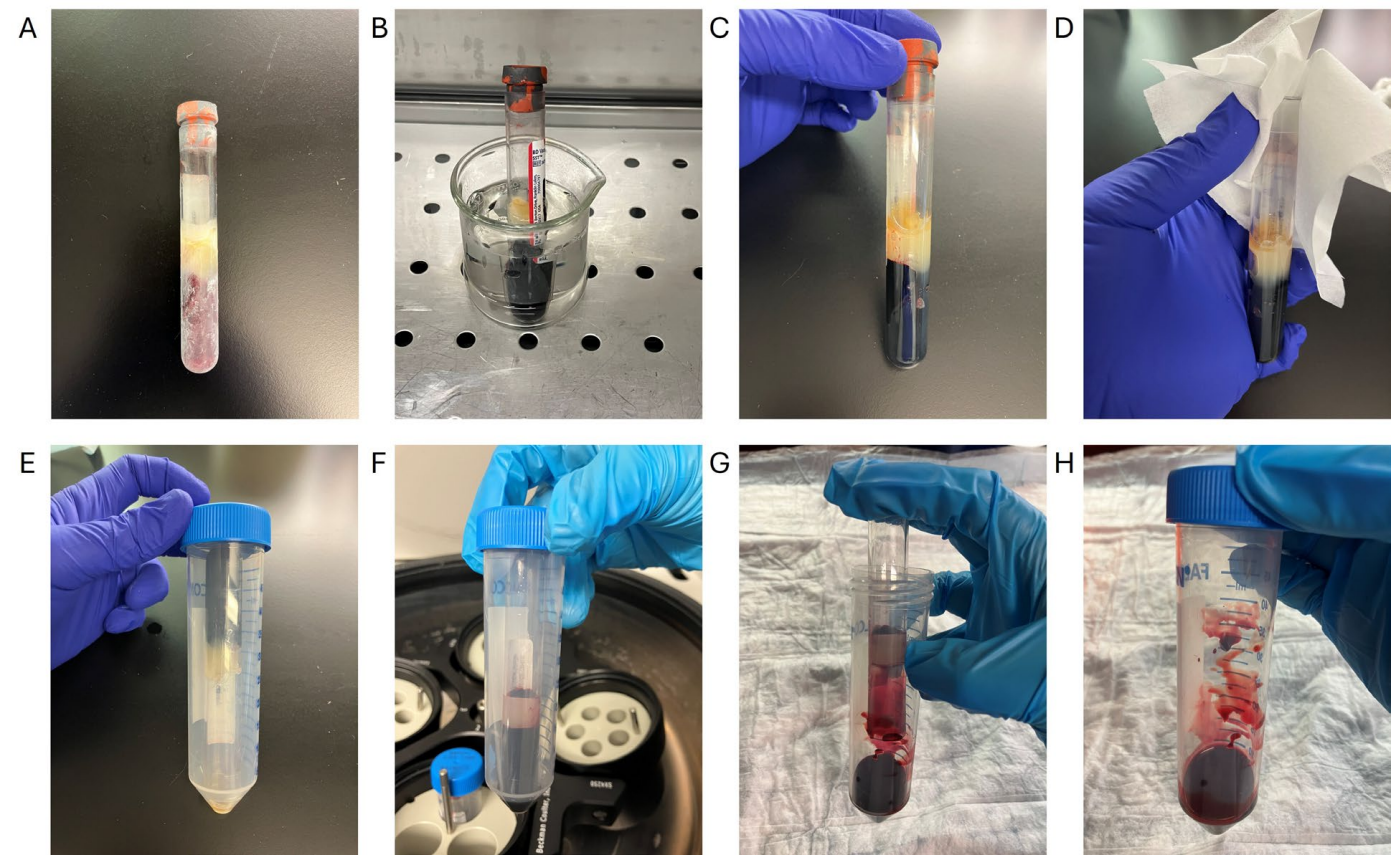
Removing a blood clot sample from a serum-separating tube. (A) Frozen blood clot. (B) Frozen blood clot is thawed at 37 °C for 3 min in the water bath. (C) Thawed blood clot. The gel layer is also thawed in this picture, and some leftover serum can be seen above this gel, indicating that this blood clot is sufficiently thawed. (D) Cleaning of the rim of the serum-separating tube using 70% ethanol. (E) The serum-separating tube is inverted inside a 50 mL conical tube. (F) Sample after initial centrifugation. The blood clot will move to be under the gel layer. (G) Removal of the serum-separating tube. The gel layer is left behind inside the serum-separating tube, leaving the blood clot inside the 50 mL conical tube. (H) Blood clot is ready for step A4 of the procedure.

1. Thaw frozen blood at 37 °C for 3 min, then transfer to ice (see Figure 1 for pictures of steps A1–3).

2. Clean the outside rim area of the tube with Kimwipes and 70% ethanol.

3. Centrifuge the serum-separating blood tube containing the blood clot upside down inside a 50 mL conical tube at 2,000 rcf for 5 min at 4 °C.

4. Put the loosened clot into a gentleMACS C tube.

5. Add 10 mL of ice-cold RBC lysis solution to rinse the 50 mL conical tube to obtain as much sample as possible using a serological pipette. Add everything to the C tube. Close the C tube tightly.

6. Run the Multi_A_01 Miltenyi protocol on the gentleMACS Octo Dissociator.

7. Vortex the tube vigorously for 3 s.

8. Place the sample on a nutator at 4 °C for 5 min.

*Note: If using fresh samples, increase this to 10 min.*

9. Pipette out the lysis solution into a new 50 mL conical tube.

10. Rinse the C tube with 5 mL of lysis buffer and then add this to the 50 mL conical tube.

11. Centrifuge at 2,000 rcf for 5 min at 4 °C to pellet the leukocytes.

*Note: It is normal for the leukocyte pellet to be reddish.*

12. Carefully discard the supernatant without disturbing the pellet.

13. Add 5 mL of RBC lysis solution to the pellet.

14. Vortex the tube vigorously for 3 s.

15. Place the sample on a nutator at 4 °C for 5 min.

*Note: If using fresh samples, increase this to 10 min.*

16. Centrifuge at 2,000 rcf for 5 min at 4 °C to pellet the leukocytes.

17. Carefully discard the supernatant.

18. Proceed to additional protocols depending on your endpoint.

a. Proceed to **section B** if flow sorting is necessary for bulk assays.

b. Skip to **section C** if performing single-nuclei assay.

c. Optional: Add a suitable volume of 90% fetal bovine serum and 10% DMSO to freeze desired aliquots of the leukocytes in cryovials for future use.

*Note: Cell count varies per individual. Aliquoting samples may not be feasible if flow sorting is necessary due to the potential low cell count per aliquot of the desired population.*

**B. Flow cytometry of lymphocytes**

1. Make MACS buffer fresh (see Recipe 2). Scale up or down depending on the number of samples. Make 10 mL per sorted population.

*Note: One blood clot tube yields a sample of B cells and T cells for a total of two sorted populations.*

2. Add 2 mL of MACS buffer to the leukocyte pellet and gently pipette up and down 3 times.

3. Put a 70 μm MACS SmartStrainer on a 5 mL conical tube and prime the strainer with 500 μL of MACS buffer.

4. Filter using a 70 μm MACS SmartStrainer in the conical tube.

5. Rinse the filter with an additional 500 μL of MACS buffer.

*Note: The total volume of the conical tube should now be 3 mL.*

6. Take out 200 μL of cell suspension and place it in a microcentrifuge tube to use as unstained controls.

7. Centrifuge at 300 rcf for 5 min.

8. Pipette out the supernatant.

9. Add 100 μL of MACS buffer to the pellet. Flick the tube gently five times to resuspend.

10. Add 1 μL of each antibody so that the dilution is 1:100. Flick the tube gently five times to mix and incubate at 4 °C for 15 min.

11. During this incubation period, make compensation bead controls by adding one drop of compensation beads and 1 μL of antibody to a microcentrifuge tube. Make a control for each antibody of interest in separate tubes.

12. Add 3 mL of MACS buffer to the stained cells.

13. Centrifuge at 300 rcf for 5 min.

14. Pipette out the supernatant and replace it with 1 mL of MACS buffer.

15. Put a 30 μm MACS SmartStrainer on a 5 mL Polyprene round-bottom tube and prime the strainer with 500 μL of MACS buffer.

16. Filter the cell suspension using a 30 μm MACS SmartStrainer.

17. Rinse the strainer with 500 μL of MACS buffer.

*Note: The total volume of the conical tube should now be 2 mL. The sample can be diluted further with additional MACS buffer if needed for flow sorting. This should be optimized for the flow cytometer device being used.*

18. Proceed to flow sorting.

*Note: See Reports S1, S2, and S3 in the Supplementary information for gating strategies.*

**C. Nuclei extraction from whole leukocytes or flow-sorted cells**

1. Add 1 mL of ice-cold lysis buffer to the cell pellet.

2. Transfer the solution to a gentleMACS C tube.

3. Rinse the conical tube containing the leftover leukocyte pellet with 1 mL of ice-cold lysis buffer. Transfer the solution into the same C tube.

4. Close the C tube tightly beyond the first resistance and place it onto the gentleMACS dissociator.

5. Place Octocoolers around the samples on the gentleMACS dissociator.

6. Run the gentleMACS program 4C_Nuclei_1 on the gentleMACS dissociator.

*Note: If using fresh samples, run this protocol twice for complete lysis.*

7. After the run, detach the C tube from the gentleMACS dissociator and immediately place it on ice.

8. Incubate on ice for 5 min (incubate for 10 min if using fresh samples).

*Note: If flow cytometry was done, skip directly to section D after this step.*

9. During this incubation, attach a MACS SmartStrainer (70 μm) to a 15 mL conical tube. Prime the strainer with 500 μL of lysis buffer.

10. Run the nuclei suspension through the primed MACS SmartStrainer (70 μm) and collect the flowthrough in a 15 mL conical tube.

11. Wash the MACS strainer with 2 mL of lysis buffer. Discard the strainer.

12. Centrifuge the nuclei suspension at 300 rcf for 5 min at 4 °C.

13. During the centrifugation step, attach a MACS SmartStrainer (30 μm) to a 5 mL conical tube. Prime the strainer with 500 μL of resuspension buffer.

14. Aspirate/pipette off the supernatant completely.

15. Resuspend the pellet with 1 mL of resuspension buffer by pipetting up and down gently 10 times.

16. Run the nuclei suspension through the primed MACS SmartStrainer (30 μm) and collect the flowthrough in a 5 mL conical tube.

17. Add 500 µL of resuspension buffer to the strainer to rinse out any remaining nuclei.

**D. Nuclei cleanup and concentration**

1. Centrifuge nucleus suspension at 300 rcf for 5 min.

2. Aspirate/pipette off the supernatant completely.

3. Resuspend the nucleus pellet in 450 μL of nuclei separation buffer.

*Note: If the entire blood clot was used instead of flow sorting, increase this volume 5 times.*

4. Add 50 μL of anti-nucleus MicroBeads.

*Note: If the entire blood clot was used instead of flow sorting, increase this volume 5 times.*

5. Mix well by gentle pipetting and incubate for 15 min at 4 °C.

6. During this incubation period, calibrate the LS columns.

7. Mount the QuadroMACS Separator Unit to the MACS Multistand.

8. Place the LS column onto the separator unit.

9. Rinse the column with 3 mL of nuclei separation buffer and allow the buffer to drip through the column. Discard the flowthrough.

10. Add 2 mL of nuclei separation buffer to the nucleus suspension from step D5.

11. Apply the nucleus suspension to the column. Apply directly to the center of the column reservoir. Discard the flowthrough.

12. Wait for the column to stop dripping before proceeding to the next step.

13. Wash the column twice with 1 mL of nuclei separation buffer. Discard the flowthrough.

14. Make sure to rinse the inner column wall with these wash steps.

15. Remove the column from the separator and place it inside a 5 mL conical tube.

16. Add 500 μL of nuclei separation buffer onto the column and immediately flush out the magnetically labeled nuclei by firmly pushing the plunger into the column.

17. Count and visualize nuclei prior to downstream assays.

## Data analysis

**Figure 2.**
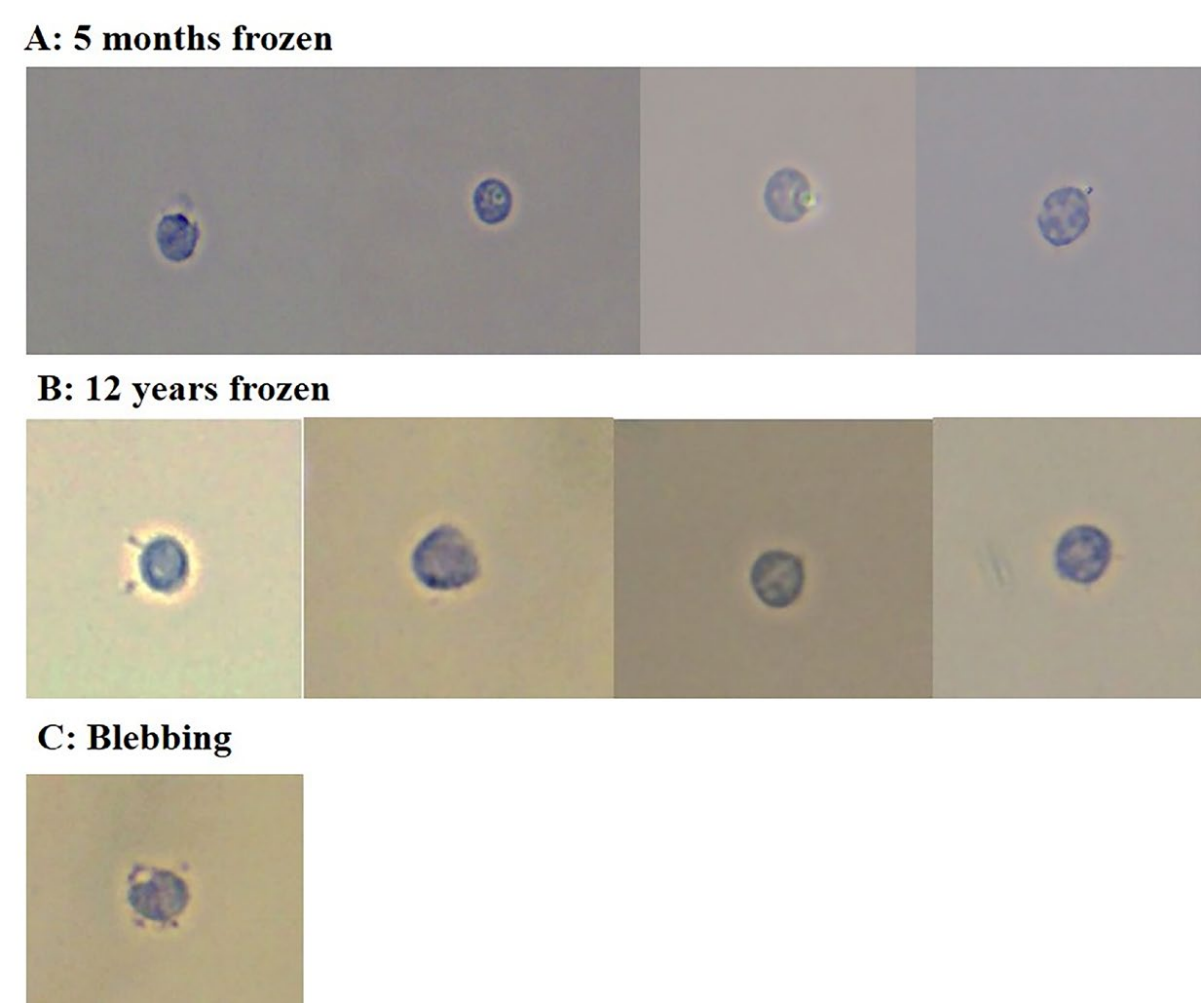
Nuclei morphology from frozen blood clots. (A) Nuclei from a blood clot frozen at -80 °C for 5 months. (B) Nuclei from a blood clot frozen at -80 °C for 12 years. The blood clot from a serum-separating tube was placed directly into the freezer without any additional manipulations. Nuclei extracted from these samples appear to be round and intact, suggesting that the nuclear envelope did not burst open. (C) Blebbing nuclei. This is an example of blebbing nuclei, which are not suitable for downstream applications. Blebbing nuclei were present in small quantities in a 12-year-old sample, but most of the nuclei extracted were intact. Nuclei were stained with 0.4% Trypan Blue prior to visualization and imaged with a brightfield microscope at 40×.

**Figure 3.**
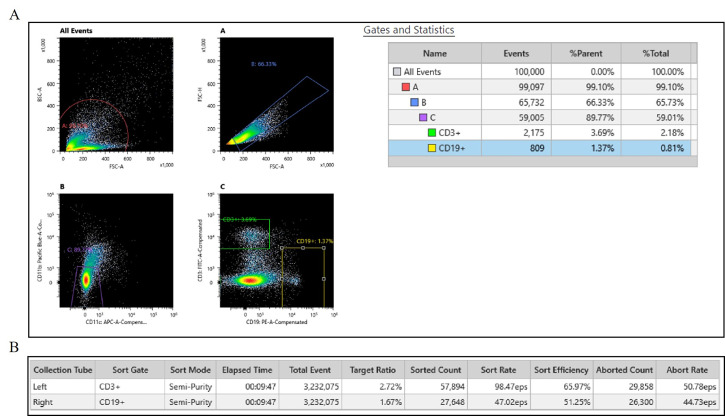
Flow cytometry results from Sony MA900 cell sorter. (A) Gating strategy for sorting singlets of B and T cells. (B) Sorted counts. Leukocytes were isolated from previously frozen blood clots and dissociated by following section A of this protocol. Cells were stained with an antibody panel targeting CD3, CD19, CD11b, and CD11c. Viability marker was not used due to interest in nuclei from subpopulations of non-viable leukocytes. After doublet exclusion and gating on height and area of cells, T-cell subsets were identified as CD3+ populations. B-cell subsets were identified as CD19+ populations. CD11b and CD11c were used to exclude monocytes. Single-stain controls were used for compensation. During this sort, we successfully sorted 57,894 T cells and 24,648 B cells. The full report is available in Supplementary material (Report S1).

**Figure 2** shows representative images of nuclei imaged after performing the extraction protocol. Blood was collected in a serum-separating tube, centrifuged at 1300× *g* for 15 min to extract serum, and the blood clot was stored immediately at -80 °C. Nuclei that are round and intact can be used for downstream assays such as bulk ATAC seq, bulk RNA seq, sn-ATAC seq, sn-RNA seq, or multiome experiments. **Figure 3** shows a representative flow cytometry gating strategy and statistics from cell sorting frozen lymphocytes without cryopreservation. Live/dead marker was not used due to the nature of the sample of interest being nuclei. Cells were gated first on BSB-H vs. SSC-H to remove debris, then SSC-A vs. SSC-H to isolate single cells, followed by CD11b and CD11c to remove monocytes (gating on the double-negative population); finally, gating was conducted on CD19- or CD3-positive to isolate B and T cells.

## Validation of protocol

We successfully performed the entirety of this protocol and yielded intact nuclei from frozen blood clots that had been frozen for 5 months. Flow cytometry was done successfully using both the Sony MA900 Multi Application Cell Sorter (see Figure 2 and Report S1) and the Miltenyi MACSQuant Tyto Cell Sorter (see Report S2 and S3) for B and T cells from frozen blood clots, suggesting the leukocyte extraction protocol is gentle on the fragile cells and cell sorting is possible despite the cells being non-viable. The yield was higher when using the Tyto sorter, likely due to the constant agitation possible within the cartridge to prevent clumping of cells (see Table 1). Nuclei morphology suggests an intact nuclear envelope without severe blebbing, as indicated by **Figure 1**. As a validation experiment, we performed bulk ATAC-seq on B and T cells obtained from sorted 5-month-old frozen blood clots. The OMNI ATAC seq library prep, outlined by Grandi et al. [18], was followed, starting from the tagmentation step through library prep. Barcoding primers used were AATGATACGGCGACCACCGAGATCTACACTGATGAAATCGTCGGCAGCGTCAGATGTGTAT index i5 + CAAGCAGAAGACGGCATACGAGATTCGCCTTAGTCTCGTGGGCTCGGAGATGTG index i7 for CD19+ cells and AATGATACGGCGACCACCGAGATCTACACGTCGGACTTCGTCGGCAGCGTCAGATGTGTAT index i5 + CAAGCAGAAGACGGCATACGAGATCTAGTACGGTCTCGTGGGCTCGGAGATGTG index i7 for CD3+ cells.

**Figure 4.**
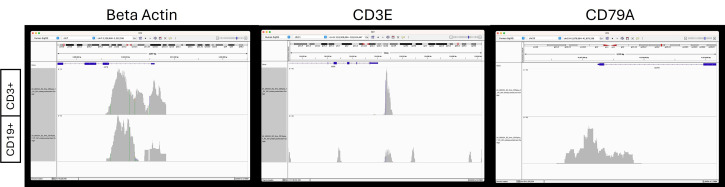
Read depths in beta actin, CD3E, and CD79A. Read depths are generous at the start site of beta actin, indicative of an accessible chromatin region for both CD3+ T cells and CD19+ B cells. For CD3E, an accessible chromatin region is present in T cells with more read depth, but not in B cells. CD79A is a known marker in B cells and has generous read depths for B cells but is absent in T cells.

As seen in **Table 2**, the library prep for bulk ATAC-seq was successful and met quality score thresholds. The “% >= Q30 bases” metric indicates that both samples are greater than the 70% threshold for ATAQ-seq. The mean quality scores of 36.86 and 37.5 are indicative of a close to 1:10,000 probability of incorrect bases called, suggesting 99.99% accuracy. Figure 4 displays read depths of open chromatin regions from nuclei extracted from flow-sorted B and T cells. These peaks are indicative of the expected open chromatin signatures of these cell types. Together, this data suggests that isolation of nuclei from frozen blood clot without any cryopreservation, as outlined in this protocol, is successful, and meaningful sequencing data can be obtained from nuclei.

**Table 1.** Flow cytometry counts from the Miltenyi MACSQuant Tyto cell sorter. Extracted leukocytes from a single blood clot sample in an 8.5 mL serum-separating tube were sorted twice to obtain B- and T-cell populations. During this sort, we successfully sorted 130,400 T cells and 61,670 B cells. The full report is available in the Supplementary materials (Reports S2 and S3).

Sample	Elapsed time	Triggered events	Gated events	Sorted events	Sort rate	Aborted events	Overall % gated	Overall % sorted
5mo_CD3+	00:14:25	234,300	131,800	130,400	150.6 eps	187	56.3%	99.9
5mo_CD19+	00:19:02	384,900	61,670	61,610	54.1 eps	62	16%	99.9

**Table 2.** Lane summary read of ATAC sequencing. Flow-sorted B and T cells were used to make two bulk ATAC-seq libraries. % >= Q30 bases metric indicates that both samples are greater than the 70% threshold for ATAQ-seq. The mean quality scores of 36.86 and 37.5 are indicative of a close to 1:10,000 probability of incorrect bases called, suggesting 99.99% accuracy.

Sample	PF clusters	% of the lane	% perfect barcode	% one mismatch barcode	Yield (Mbases)	% PF clusters	% >= Q30 bases	Mean quality score
5mo_CD19+	89,025,951	6.39	94.47	5.53	26,886	100	85.13	36.86
5mo_CD3+	34,629,353	2.48	96.12	3.88	10,458	100	88.19	37.5

Overall, these results are indicative that extracting nuclei from previously frozen samples with no cryopreservation is possible and may be of great interest for labs that have existing biobank samples. The addition of flow cytometry opens up the possibility of meaningful bulk assays that may be more cost-effective; however, we anticipate that this protocol may also be suitable for single-nuclei assays. It is important to note that due to the lack of cryopreservation, causing non-viability of the cells, this protocol should only be coupled with downstream nuclei assays, not single-cell assays.

## General notes and troubleshooting

**General notes**

Leukocyte count can vary significantly between individuals depending on numerous health factors. This protocol was validated using 3 mL of blood clots, but the volumes of reagents can be scaled up or down depending on the sample amount.

This protocol was developed and validated using frozen human blood clot; however, we anticipate this may be appropriate to use in blood samples of other species.

**Troubleshooting**

1. Sample is clumpy after leukocyte dissociation: This is normal; clumps will break down after running through several cell strainers in subsequent steps of the protocol.

2. Incomplete lysis: Depending on the initial handling of the blood samples, lysis times may need optimization. We found that 5 min on the nutator was sufficient for frozen samples, but fresh samples required longer incubation time. Incomplete lysis will be apparent by Trypan Blue staining and visualization with a microscope, marked by the presence of cells (unstained) as opposed to nuclei (stains blue).

## Supplementary information

The following supporting information can be downloaded here:

1. Report S1. Sony MA900 Flow Sorting Report

2. Report S2. MACSQuant Tyto Run Report B cell

3. Report S3. MACSQuant Tyto Run Report T cell
